# Sulfur-enriched leonardite and humic acid soil amendments enhance tolerance to drought and phosphorus deficiency stress in maize (*Zea mays* L.)

**DOI:** 10.1038/s41598-020-62669-6

**Published:** 2020-04-14

**Authors:** Cengiz Kaya, Mehmet Şenbayram, Nudrat Aisha Akram, Muhammed Ashraf, Mohammed Nasser Alyemeni, Parvaiz Ahmad

**Affiliations:** 1 Harran University, Faculty of Agriculture, Department of Soil Science & Plant Nutrition, Şanlıurfa, Turkey; 2 Department of Botany, GC University Faisalabad, Faisalabad, Pakistan; 3 University of Agriculture Faisalabad, Faisalabad, Pakistan; 4 Botany and Microbiology Department, College of Science, King Saud University, Riyadh, Saudi Arabia; 5 Department of Botany, S.P. College Srinagar, Srinagar, Jammu and Kashmir India

**Keywords:** Abiotic, Drought

## Abstract

Soil amendments are known to promote several plant growth parameters. In many agro-ecosystems, water scarcity and drought induced phosphorus deficiency limits crop yield significantly. Considering the climate change scenario, drought and related stress factors will be even more severe endangering the global food security. Therefore, two parallel field trials were conducted to examine at what extent soil amendment of leonardite and humic acid would affect drought and phosphorus tolerance of maize. The treatments were: control (C: 100% A pan and 125 kg P ha^−1^), P deficiency (phosphorus stress (PS): 62.5 kg P ha^−1^), water deficit stress (water stress (WS): 67% A pan), and PS + WS (67% A pan and 62.5 kg P ha^−1^). Three organic amendments were (i) no amendment, (ii) 625 kg S + 750 kg leonardite ha^−1^ and (iii) 1250 kg S + 37.5 kg humic acid ha^−1^) tested on stress treatments. Drought and P deficiency reduced plant biomass, grain yield, chlorophyll content, *F*_v_*/F*_m_, RWC and antioxidant activity (superoxide dismutase, peroxidase, and catalase), but increased electrolyte leakage and leaf H_2_O_2_ in maize plants. The combined stress of drought and P deficiency decreased further related plant traits. Humic acid and leonardite enhanced leaf P and yield in maize plants under PS. A significant increase in related parameters was observed with humic acid and leonardite under WS. The largest increase in yield and plant traits in relation to humic acid and leonardite application was observed under combined stress situation. The use of sulfur-enriched amendments can be used effectively to maintain yield of maize crop in water limited calcareous soils.

## Introduction

The global climate change simulations suggest fresh water availability will further deplete in many rainfed and irrigated agricultural areas^1^ and thus, threatens food security^2^. Although hydrological, meteorological and agricultural droughts occur simultaneously and are interrelated with each other, agricultural drought is believed to be the most common^3,4^. Water stress causes a variety of responses from physiological to molecular in plants, allowing them to acclimate to harsh ecological conditions^5^. Drought susceptibility of plants differs according to the plant species, stress level, and growth stages^6^.

Considerable yield gaps have been noticed in agricultural systems^7,8^ and the availability of good quality water and mineral nutrients is critical for overcoming these yield gaps^9–11^. This is principally reasonable for maize crop, one of the main cereals of the globe, covering 26% and 37% of the total cereal cultivated area and production, respectively^12^. Maize is known as one of the highest water-requiring crops. Water deficiency imposed at any stage of its development can reduce grain yield significantly^13,14^. As it is a fast-growing crop, its requirement for essential nutrients is also high and deficiency of any of the plant nutrients may lead to hamper growth and decrease yield^15^. Maize is particularly susceptible to P deficiency, which can suppress growth and grain yield^16–18^.

Phosphorus (P) has been reported to be one of the limited mineral elements in most agro-ecosystems^19,20^. This element is involved in a number of key energy transfer and photosynthetic oxidation-reduction reactions^21^. Phosphorus is also part of a broad range of biochemical compounds including nucleic acids, structural proteins and enzymes as well as signal transductions^22,23^. The soil available P for plants is frequently insufficient because of its strong binding in insoluble forms^24,25^. Plants have developed strategies to alleviate P deficiency^26^, which include increased efflux of organic acids^27^, altered root structure^28^, and enhanced acid phosphatase activity^29^. All these mechanisms contribute to increased P intake in plants under P-deficient regimes^23^.

Organic fertilizer use has gained a great attention as a means to improve crop nutrition and soil fertility. Organic fertilizers have a main function in improving the quantity of organic matter in the root zone^30^. Leonardite is one of the organic matters with high P content available for this purpose^31^. Leonardite either is an oxidation product of lignite related to subsurface mining^32,33^ or it consists of sediments enriched with humic compounds^34,35^. It contains a high quantity of humic substances (from 20% to more than 70%). Humic compounds present in the soil affect directly or indirectly plant growth^36^. Humic acid application leads to an increase in some key plant biochemicals such as nucleic acids, vitamins, amino acids, and nutrients, but it also improves soil chemical properties^36,37^.

Like phosphorus, sulfur (S) is known to be one of the most crucial major nutrients essential for plant growth^38–40^. Sulfur plays a role in the building of proteins and chlorophyll^41–43^. Sulfur deficient regimes suppress cell sap osmotic pressure, which is ascribed to limited accumulation of intracellular solutes^44^. Under S deficit conditions, it is known that SO_4_^2−^ is mobilized to sustain plant growth, and that its contribution to osmotic adjustment is compensated by other osmotically active molecules^45^.

Many authors have depicted responses of crops to either phosphorus (P) deficiency^17,46–48^ or water stress^49–53^, but not much research has been carried out so far to appraise the combined effects of P deficiency and water stress on crops^54^. Furthermore, no sufficient literature exists on the role of sulfur-enriched leonardite and humic acid in maize plants subjected to the combined application of both stresses. In the current study, it was aimed to assess the possible effects of these soil amendments in maize plants subjected to P deficiency and water deficit conditions applied singly or jointly.

## Material and Methods

### Field conditions

Two parallel field trials were conducted in 2011 at the Agricultural Research Station, University of Harran, Sanliurfa, Turkey, during the appropriate maize growth season (end-June to end-October). Soil samples taken randomly from the top 0–30 cm horizon of the experimental field before planting were analyzed according to Ryan, *et al*.^55^. The texture of the soil used was clay loam with pH of 7.8, CaCO_3_ 25.2%, organic matter content 1.2%, available P 20 kg ha^−1^ [0.5 *M* NaHCO_3_ extractable P_2_O_5_^56^], plant-available S 3.1 µg g^−1^ [0.01 *M* CaCl_2_ extractable SO_4_^−2^-S^57^] and electrical conductivity of saturation extract 1.1 dS m^−1^. The soil had field capacity 32.6%, permanent wilting point 25.6% and dry bulk density 1.37 g cm^−3^. The exchangeable cation contents of K^+^, Ca^2+^, Mg^2+^ and Na^+^ were 1.35, 25.5, 12.2 and 0.67 cmol kg^−1^, respectively. Electric conductivity and pH of the water used for irrigation were 0.53 dS m^−1^ and pH 7.3, respectively.

During the trial, total rainfall and average relative humidity were 3.8 mm and 30.3%, respectively. Maximum, minimum and mean of temperature (°C) during the experiment were 38.7, 18.9 and 29.4, respectively. Weed infestation was controlled manually three times during the season.

Evaporation was appraised using a Class A Evaporation Pan situated close to the field plots for manual measurement of daily evaporation. The volume of water used during each irrigation was calculated following the class A pan evaporation using the below given formula^58^:

$$Ir=Epan-Kcp$$

where *Ir* is the amount of irrigation water used (mm), *Epan* is the cumulative evaporation at class A pan between two irrigations, and *Kcp* is the plant-pan coefficient.

### Experimental design

The experimental design comprised four stress treatments: control (C:100% A pan and 125 kg P/ha), P deficiency (PS: 100% A pan and 62.5 kg P/ha), water deficit stress (WS: 67% A pan and 125 kg P/ha), and PS + WS (67% A pan and 62.5 kg P/ha). In the water stress treatment, plants were irrigated every three days at 67% A pan (Epan) evaporation, whereas the control plants received 100% A pan every day. Phosphorus in the form of superphosphate was incorporated into the soil prior to sowing. According to the findings of a pilot glasshouse trial using a series of concentrations of sulfur (S) and leonardite (LEO) applied individually or in combination, two combinations of soil amendments (SA) (SA1: 625 kg S + 750 kg LEO/ha and SA2: 1250 kg S plus 37.5 kg humic acid/ha) were selected for the present field trial. Leonardite and liquid humic acid were provided by Biotar Company (Ankara, Turkey). Before using these soil amendments, available P was analyzed in order to know whether or not any significant amount of P was released from them into the soil. Leonardite and humic acid contained 350 and 5 mg available P kg^−1^ which can supply maximum 262.5 and 0.18 g of P from leonardite and humic acid, respectively, based on their application rates per ha. So such amount of P released from LEO and HA could be considered as insignificant compared to the amount of P applied. The trial was designed in a randomized split plot design (stress treatments as main plots, soil amendments as sub-plots). All trial units were replicated thrice. Ninety-six plants of maize (cv. DKC-5789) per treatment were maintained in a planting geometry of 0.25 m plant to plant distances and 0.7 m row to row distance within each experimental unit of 6.0 m × 2.8 m.

All plots were drip-irrigated (4 L h^−1^ m^−1^ from 10:00 am to 5:00 pm for two weeks) for a good establishment of seedlings. The drip system operating pressure was fixed at 100 kPa during the entire growth period. For irrigating each row, a single drip tube with 0.5 m emitter spacing was positioned on the surface of soil. For precisely monitoring the schedule of irrigation intervals, soil tensiometers at 30 and 45 cm soil depths centering between two plants in a row were installed. The tensiometer readings were maintained above the threshold level, i.e., −30 kPa and −20 kPa at 30 and 45 cm depth, respectively, for well-watered plants, and −65 kPa and −50 kPa at 30 and 45 cm depths, respectively, for the water stress treatment.

Nitrogen (urea) and potassium (potassium sulphate) fertilizers at the rate of 200 N and 240 K_2_O kg ha^−1^ were applied to each experimental unit. Potassium fertilizer was broadcast in the soil before planting the crop, while the urea was applied in an equal dose through the drip irrigation three times at two-week intervals.

### Plant measurements

Youngest completely extended 3rd leaf from the apex was collected at dawn from each of 10 plants selected randomly from one of 4 rows for the quantity of leaf relative water content (RWC), electrolyte leakage (EL), chlorophyll content, malondialdehyde and hydrogen peroxide contents, acid phosphatase activity, hydrogen peroxide content and maximal quantum yield.

Leaf RWC was assessed using the method of Kaya, *et al*.^59^ adopted from Yamasaki and Dillenburg^60^. Leaf EL was determined using the procedure developed by Dionisio-Sese and Tobita^61^. A fresh leaf sample (200 mg) was cut into about 5 mm pieces and kept them in a glass test tube containing 10 mL of distilled water. The sample was retained for 60 min at 32 °C in a water bath, and electrical conductivity (EC_1_) was quantified and then the materials were kept in an autoclave at 121 °C for 20 min. The sample solution was cooled down to 25 °C and EC of the solution re-measured (EC_2_). The equation followed for determining the membrane permeability was: MP = EC_1_/EC_2_ × 100.

Chlorophyll content was determined by using 1.0 g of recently expanded leaf, which was triturated in 90 percent acetone solution. The absorbance of the filtrate was recorded using a UV-visible spectrophotometer (Shimadzu UV-120, Japan) and the chlorophyll pigment was quantified according to Strain and Svec^62^. For enzymes, MDA and hydrogen peroxide assays, the leaf tissues were stored in liquid nitrogen at −80 °C. The activity of acid phosphatase was assessed based on the procedure given by Kaya, *et al*.^59^, adopted from Besford^63^. For antioxidant enzyme analysis, a fresh leaf tissue (500 mg) was ground in sodium phosphate buffer (50 mM pH 7.0) consisting of polyvinyl pyrolidine (1%). The material was centrifuged at 10,000 *g* for ¼ h at 4 °C and the supernatant was used for appraising the activities of catalase^64^, superoxide dismutase^65^ and peroxidase^66^. Malondialdehyde (MDA) content, as a measure of lipid peroxidation, was appraised as described by Weisany, *et al*.^67^. Hydrogen peroxide (H_2_O_2_) content was assayed as depicted in Loreto and Velikova^68^. For the measurement of maximum potential quantum efficiency of photosystem II (*F*_*v*_*/F*_*m*_) previously dark adapted leaves (for 30 min) were subjected to a portable chlorophyll fluorometer (Photosynthesis Yield Analyzer Mini-PAM, Walz, Germany).

At the initiation of grain filling phase, three plants from each of 4 rows were harvested from the ground level to determine above ground shoot fresh weight. The shoots from three plants randomly selected from each experimental unit were dried for 2 days at 70 °C to appraise above ground shoot dry weight.

At day 120 after sowing, 24 plants chosen randomly from the remaining three rows were harvested from each experimental unit. The cobs were removed from the stalks and grain yield assessed at 13% moisture content. Thousand-grain weight (TGW) was also recorded. The dried plant material was processed by ashing at 550 °C for 6 h in a muffle furnace. The concentration of phosphorus was assayed by employing the Vanadate-molybadate method^69^.

### Statistical analysis

Statistical analysis of the data gathered from the two parallel experiments was carried out. The two individual experiments did not differ significantly in terms of the analysis of variance of the data, so the data of the two experiments were averaged. Significant differences among the mean values were appraised using the Duncan’s test at 5% probability level. Data in all Figures are presented as mean ± standard error.

## Results

### Sulfur-enriched soil amendments improve plant growth and grain yield under phosphorus deficiency and water stress

**Figure 1:**
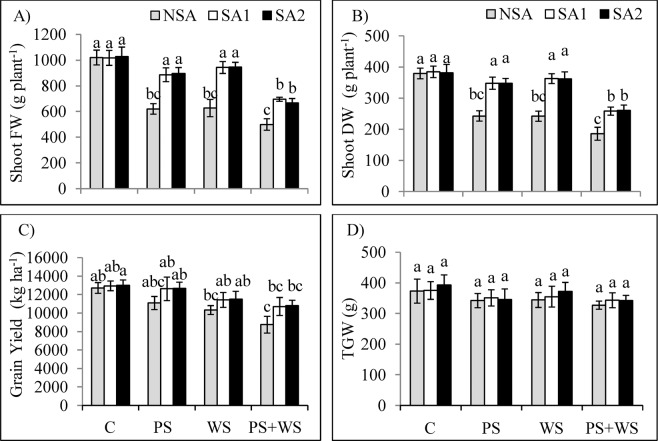
Shoot fresh weight [FW; (**A**)] and shoot dry weight [DW; (**B**)], grain yield (**C**) and thousand grain weight [TGW; (**D**)] of maize plants grown under phosphorus and water deficiency stresses applied individually or in combination with or without soil amendments (SA1 and SA2). Mean ± S.E.; Mean pairs with different letters are significantly different (*P* < 0.05) by Duncan’s multiple range test. (**C**) Control treatment (Well-watered; plants were irrigated once every day days at 100% A pan (Epan) evaporation, and adequate P, 125 kg P/ha); PS: phosphorus deficiency stress (62.5 kg P/ha); WS: water stress (Plants were irrigated once every 3 days at 67% A pan (Epan) evaporation); NSA: No soil amendment; SA1: 625 kg sulfur (S) + 750 kg leonardite/ha; SA2: 1250 kg S + 37.5 kg/ha humic acid.

Phosphorus deficiency stress (PS) and water stress (WS) significantly decreased shoot fresh weight (39.2 and 38.6%, respectively), shoot dry weight (35.9 and 36.1%, respectively) and grain yield (12.8 and 18.75%) respectively, but did not affect thousand grain weight (Fig. 1A–D). The combined effect of drought stress and P deficiency caused a significant yield deprivation, in which the decrease in shoot DM, and grain yield were 50.1 and 31.3% relative to those in non-stressed plants, respectively. Application of soil amendments in SA, SA1 and SA2 treatments enhanced the yield parameters significantly in all stress treatments with the exception of thousand grain weight (TGW). The effect of leonardite and humic acid treatment on yield parameters were less significant in the combined stress treatment (PS + WS) compared to that by the single stress treatment. Both additives (humic acid and leonardite) positively affected the yield parameters with no significant difference among them.

### Sulfur-enriched soil amendments improve leaf total chlorophyll content and maximum fluorescence yield under phosphorus deficiency and water stress

**Figure 2:**
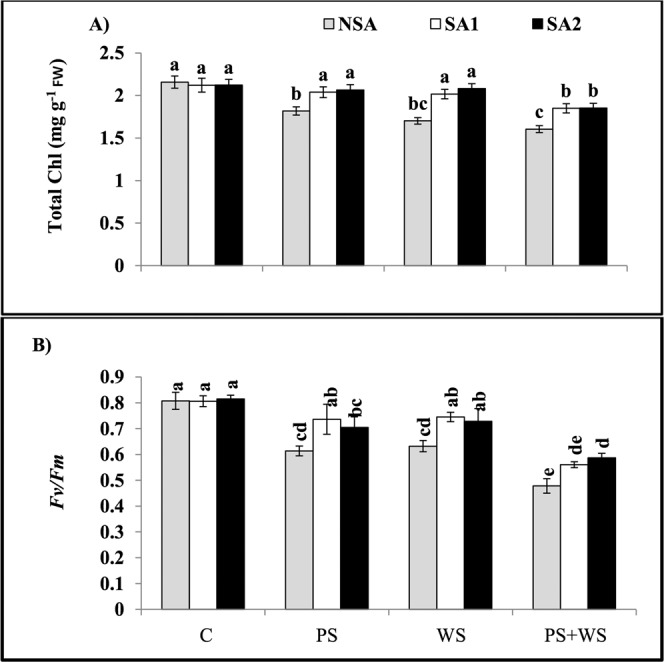
Leaf total chlorophyll contents (**A**) and maximum fluorescence yield **[***F*v*/F*m; (**B**)**]** of maize plants grown under phosphorus and water deficiency stresses applied individually or in combination with or without soil amendments (SA1 and SA2). Mean ± S.E.; Mean pairs with different letters are significantly different (*P* < 0.05) by Duncan’s multiple range test. (**C**) Control treatment (Well-watered; plants were irrigated every day at 100% A pan (Epan) evaporation, and adequate P, 125 kg P/ha); PS: phosphorus deficiency stress (62.5 kg P/ha); WS: water stress (Plants were irrigated once every 3 days at 67% A pan (Epan) evaporation); NSA: No soil amendment; SA1: 625 kg sulfur (S) + 750 kg leonardite/ha; SA2: 1250 kg S + 37.5 kg/ha humic acid.

When P deficiency and water stress applied alone, leaf total chlorophyll contents (16% and 21%, respectively) and maximum fluorescence yield (*F*_v_/*F*_m_) (24% and 21%, respectively) decreased significantly compared to those in the the control treatment (Fig. 2A,B). Furthermore, combination of both stress factors led to a greater decrease in the total chlorophyll content and *F*_v_/*F*_m_ by 26 and 41% relative to those in the non-stressed plants, respectively. Application of soil amendments (SA, SA1 and SA2) significantly enhanced total chlorophyll content by 12.1 and 13.7% and *F*_v_/*F*_m_ by 19.9 and 14.8%, respectively compared to those in the P deficient treatment (PS). These increases in leaf total chlorophyll content and *F*_v_/*F*_m_ were 18.8 and 17.9% in the S1 treatment, and 22.3 and 15.2% in the S2 treatment compared to those in the WS treatments. Application of SAs was not effective in altering these plant traits when plants were subjected to both stresses. Similarly, application of SAs did not affect these parameters in the control plants.

### Sulfur-enriched soil amendments improve leaf water potential and leaf relative water content under phosphorus deficiency and water stress

**Figure 3:**
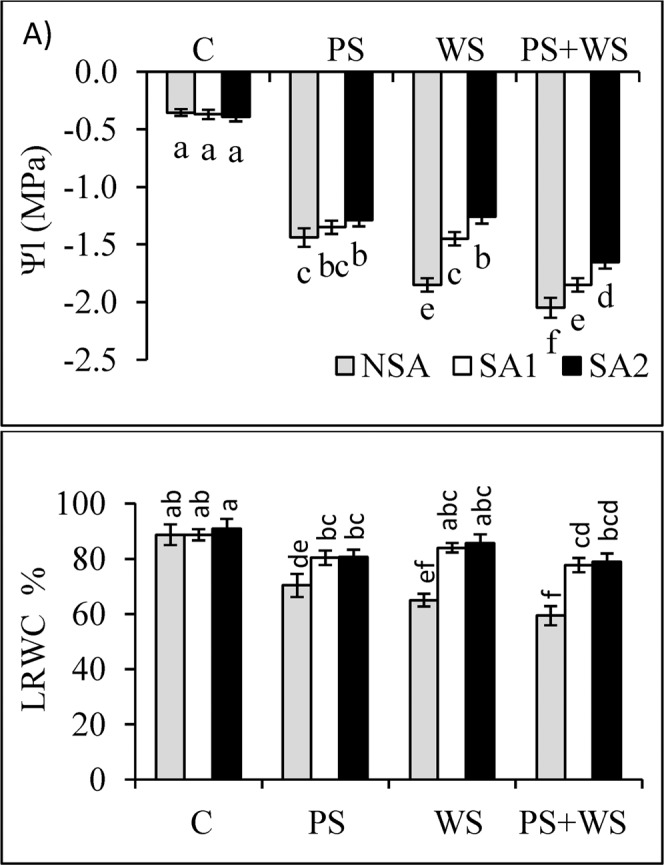
Leaf water potential **[**Ψl; (**A**)] and leaf relative water content [LRWC; (**B**)] of maize plants grown under phosphorus and water deficiency stresses applied individually or in combination with or without soil amendments (SA1 and SA2). Mean ± S.E.; Mean pairs with different letters are significantly different (*P* < 0.05) by Duncan’s multiple range test. (**C**) Control treatment (Well-watered; plants were irrigated every day at 100% A pan (Epan) evaporation, and adequate P, 125 kg P/ha); PS: phosphorus deficiency stress (62.5 kg P/ha); WS: water stress (Plants were irrigated once every 3 days at 67% A pan (Epan) evaporation); NSA: No soil amendment; SA1: 625 kg sulfur (S) + 750 kg leonardite/ha; SA2: 1250 kg S + 37.5 kg/ha humic acid.

Leaf water potential (Ψl) and leaf relative water content (LRWC) decreased by 31.1% and 20.6% in the PS treatment and by 42.8% and 26.7% in the WS treatment, and by 48.5% and 33.1% in the PS + WS treatment (Fig. 3A,B**)**. Application of SAs (in the SA1 and SA2 treatments) improved leaf Ψl by 6.2% and 10.4% and LRWC by14.2% and 14.7% in plants exposed to P deficiency. Similarly, application of SAs also improved both leaf Ψl and LRWC in plants treated with water stress. The increase in Ψl and LWRC due to the SAs application in the combined stress treatment (PS + WS) was 9.8% and 30.8% in SA1 and 19.5% and 33.2% in SA2 treatments, respectively. In most cases, there were no statistical differences between the two exogenous applications of SAs (SA1 and SA2) with respect to the water relation parameters. Similarly, no significant effects were observed in these parameters by the application of SAs to the control plants.

### Sulfur-enriched soil amendments maintain leaf P and acid phosphatase enzyme activity under phosphorus deficiency and water stress

**Figure 4:**
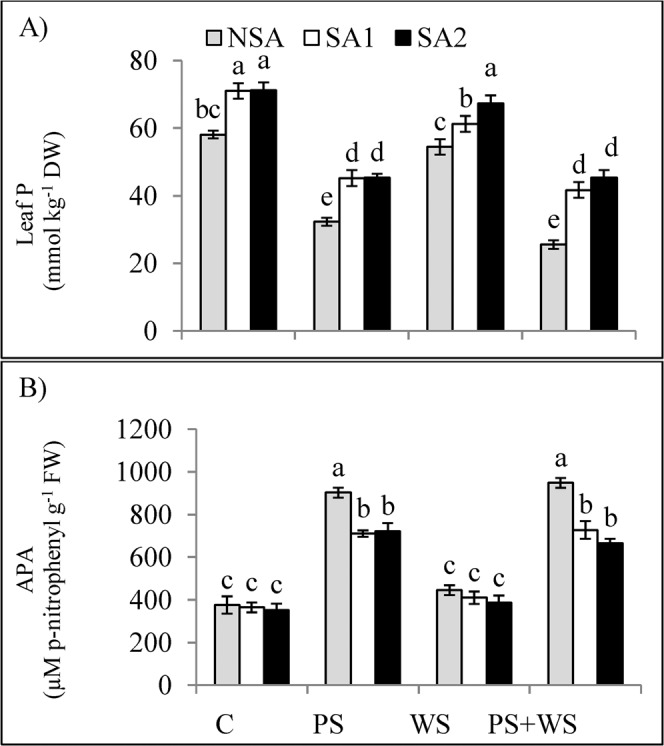
Leaf phosphorus **[**P; (**A**)] concentration and leaf acid phosphatase **[**APA; (**B**)] of maize plants grown under phosphorus and water deficiency stresses applied individually or in combination with or without soil amendments (SA1 and SA2). Mean ± S.E.; Mean pairs with different letters are significantly different (*P* < 0.05) by Duncan’s multiple range test. (**C**) Control treatment (Well-watered; plants were irrigated every day at 100% A pan (Epan) evaporation, and adequate P, 125 kg P/ha); PS: phosphorus deficiency stress (62.5 kg P/ha); WS: water stress (Plants were irrigated once every 3 days at 67% A pan (Epan) evaporation); NSA: No soil amendment; SA1: 625 kg sulfur (S) + 750 kg leonardite/ha; SA2: 1250 kg S + 37.5 kg/ha humic acid.

Leaf P content decreased by 80%, but leaf acid phosphatase enzyme activity (APA) increased by 2.4-fold in non-SAs applied P deficient treatment compared to the control plants (Fig. 4A,B. On the other hand, leaf P content and APA remained unaffected under water stress. In the combined stress treatment, however, leaf P content and APA followed the trend as observed in the P deficient treatment. Application of SAs showed positive effects on leaf P content in all treatments. On the other hand, the effect of SAs was negative on APA in all stress treatments.

### Sulfur-enriched soil amendments reverse oxidative stress parameters under phosphorus deficiency and water stress

**Figure 5:**
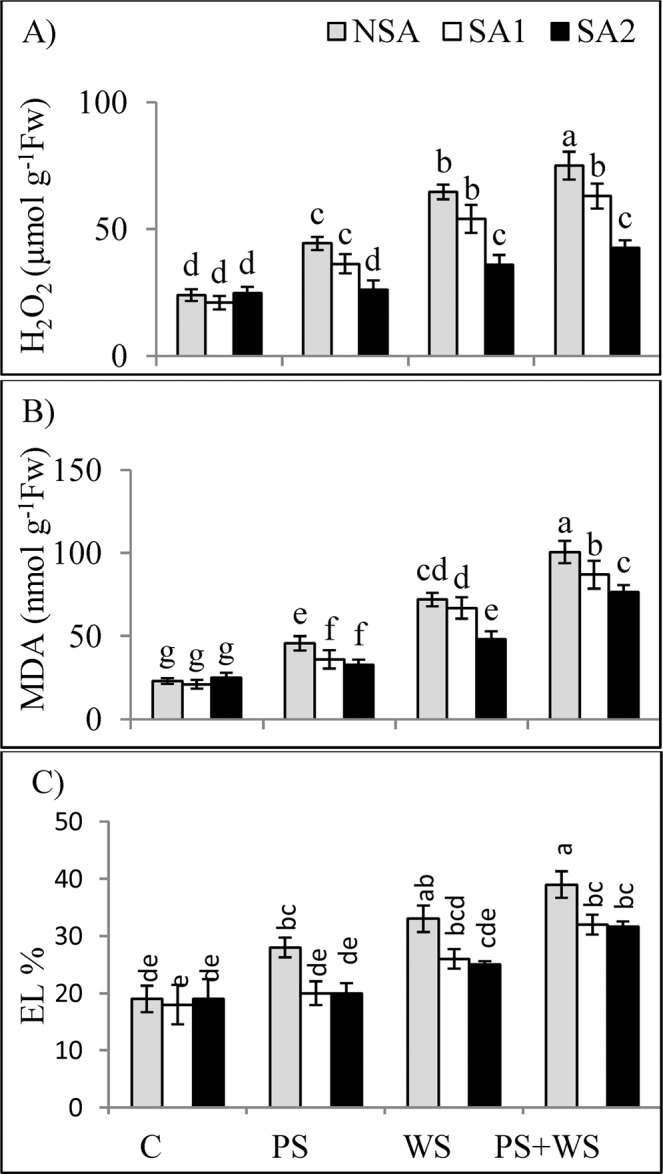
Leaf hydrogen peroxide **[**H_2_O_2_; (**A**)], malondialdehyde [MDA; (**B**)] and electrolyte leakage [EL; (**C**)] of maize plants grown under phosphorus and water deficiency stresses applied individually or in combination with or without soil amendments (SA1 and SA2). Mean ± S.E.; Mean pairs with different letters are significantly different (*P* < 0.05) by Duncan’s multiple range test. (**C**) Control treatment (Well-watered; plants were irrigated every day at 100% A pan (Epan) evaporation, and adequate P, 125 kg P/ha); PS: phosphorus deficiency stress (62.5 kg P/ha); WS: water stress (Plants were irrigated once every 3 days at 67% A pan (Epan) evaporation); NSA: No soil amendment; SA1: 625 kg sulfur (S) + 750 kg leonardite/ha; SA2: 1250 kg S + 37.5 kg/ha humic acid.

Malondialdehyde (MDA) and hydrogen peroxide (H_2_O_2_) contents as well as electrolyte leakage (EL) increased significantly under P deficiency and water stress. Here the effect was more pronounced when the stress was applied in combination (Fig. 5A–C). The content of MDA and EL decreased significantly when SAs was applied with the effect being more significant in the SA2 compared to those in the SA1 treatments under water stress alone and in combined stress situation. Similarly, a greater decline in H_2_O_2_ level was noticed in the SA2 compared to the SA1 treatments in all stress treatments. In the control plants, application of SAs did not have any significant impact on the oxidative stress parameters measured.

### Sulfur-enriched soil amendments improve antioxidant defence system under phosphorus deficiency and water stress

**Figure 6:**
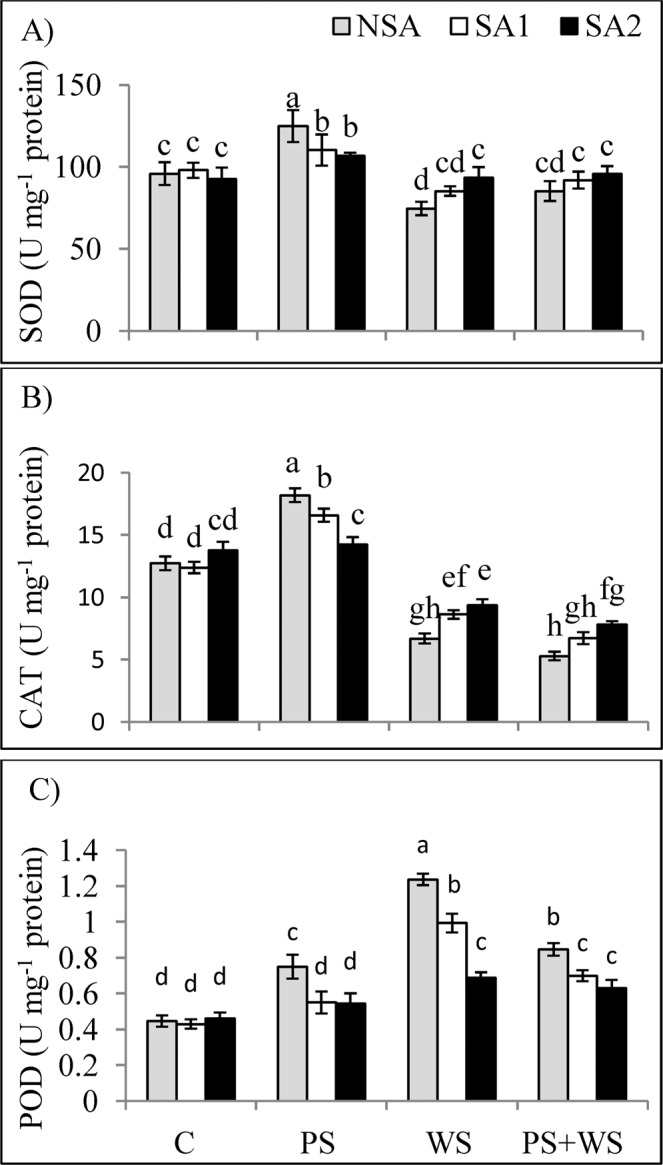
Activities of superoxide dismutase **[**SOD; (**A**)], catalase **[**CAT; (**B**)] and peroxidase **[**POD; (**C**)] in the leaves of maize plants grown under phosphorus and water deficiency stresses applied individually or in combination with or without soil amendments (SA1 and SA2). Mean ± S.E.; Mean pairs with different letters are significantly different (*P* < 0.05) by Duncan’s multiple range test. (**C**) Control treatment (Well-watered; plants were irrigated every day at 100% A pan (Epan) evaporation, and adequate P, 125 kg P/ha); PS: phosphorus deficiency stress (62.5 kg P/ha); WS: water stress (Plants were irrigated once every 3 days at 67% A pan (Epan) evaporation); NSA: No soil amendment; SA1: 625 kg sulfur (S) + 750 kg leonardite/ha; SA2: 1250 kg S + 37.5 kg/ha humic acid.

Phosphorus deficiency enhanced the activities of superoxide dismutase (SOD), catalase (CAT) and peroxidase (POD) enzymes by 30.2, 42.9 and 66.6% compared to those in the control treatment, respectively. Water deficit conditions suppressed SOD and CAT activities by 22.2 and 47.2%, but increased POD activity by 2.9-fold compared to those in the control plants, respectively. However, when combined stress was applied, CAT activity decreased by 58.4%, but POD increased by 89.5% compared to the controls (Fig. 6A–C). Under P deficiency, application of SAs significantly decreased SOD, CAT and POD activities. However, under water stress, SAs led to an elevation in SOD and CAT activities, but a reduction in POD activity. In the combined stress treatments, POD activity decreased, but the CAT activity increased significantly when SAs was applied.

## Discussion

### Effect of phosphorus deficiency, water stress and their combination and S-enriched amendments on growth and yield attributes

In our study, single or combined P deficiency and water stress reduced growth and grain yield of maize plants. Nutritional imbalance is one of the major drought-induced disturbances. Among essential nutrients, P is a crucial macronutrient that is mainly responsible for the energy balance of the higher plants. Phosphorus deficiency in soil does not only hamper P uptake and accumulation, but also may limit the uptake of other nutrients, particularly of Mg and K^70^. It is well known that an optimum amount of water as well as essential nutrients including P and S are required to attain optimal yield^71–73^.

Many investigations have stated alleviating effects of humic acid and sulfur on crop growth. Humic acid (HA) is contemplated as an important bio-stimulator that improves photosynthesis, respiration, permeability of cell membranes, and uptake of phosphate and potassium, as well as contributes to maintain hormonal balance^74^. It also improves the fertility of soil by improving its biological, physical and chemical properties^75–77^. Nakasha, *et al*.^78^ observed that application of HA before planting of safed musli (*Chlorophytum borivilianum* L.) at the rate of 5, 10, and 15% not only enhanced tuber sprouting, but hastened uniform sprouting pattern, and increased leaf area index, leaf area, number of leaves, and total root length. Similarly, S is an essential plant nutrient needed for maintaining optimal plant growth. It is available usually in the form of anionic sulfate^79^. The positive effect of sulfur on crop yield and growth has also been reported in different studies. For instance, Sanli, *et al*.^80^ reported a marked rise in tuber yield production of potato by exogenously applied leonardite at 400 kg ha^−1^. For example, Govahi and Saffari^81^ observed in canola (*Brassica napus* L. var. *oleifera*) that dry-matter accumulation increased with the addition of S at a rate of 40 to 120 kg ha^−1^. However, the optimum dose of sulfur causing a maximal change in growth differs from crop to crop. For example, sulfur application at the rate of 4 mM enhanced vegetative growth, while high concentration (8 mM) delayed the vegetative growth of onion plants^82^. Little information, however, exists until now on the effects of S-enriched humic acid application on crop productivity. Our results indicate that exogenously applied S-enriched leonardite (SA1 and SA2) is an effective chemical for improving plant growth and yield of maize plants subjected to P deficiency, water stress or under the combination of both. So, application of S-enriched SAs including leonardite or humic acid might be a quite effective means to surmount the deleterious effects of phosphorus deficiency and water stress on plants by improving phosphorus nutrition, water relations and antioxidant defense system, as well as reducing oxidative stress, which have been discussed in detail in the latter sections.

### Effect of phosphorus deficiency, water stress and their combination, and S-enriched amendments on chlorophyll contents, and membrane and PSII integrity

Photosynthetic pigments such as chlorophyll are the key components for carrying out light reactions of photosynthesis. These pigments are very fragile, and their ultrastructure and functioning are considerably impaired under stress situations including drought stress^83,84^. In the current experiment, total chlorophyll content was markedly reduced in the maize crop under water stress and P deficiency, but even more severe effect was observed when both stresses were combined. Previous research reports have shown that the stress-induced decline in chlorophyll content was primarily ascribed to considerable accumulation of H_2_O_2_ in the plant leaves^85,86^. So, the decreased total chlorophyll content might be linked to considerable accumulation of H_2_O_2_ in the maize plant leaves as observed under P deficiency and water stress. Application of SAs specifically in SA2 treatment significantly reduced the H_2_O_2_ level of the leaf and elevated the total chlorophyll content. Drought-induced decline in chlorophyll pigments has also been reported in different crops in different studies e.g. canola by Akram, *et al*.^87^, quinoa (*Chenopodium quinoa* Willd.) by Aziz, *et al*.^88^, wheat (*Triticum aestivum* L.) by Kosar, *et al*.^89^ and Huseynova, *et al*.^90^, and carrot (*Daucus carota* L. subsp. *sativus*) by Razzaq, *et al*.^91^.

**Figure 7:**
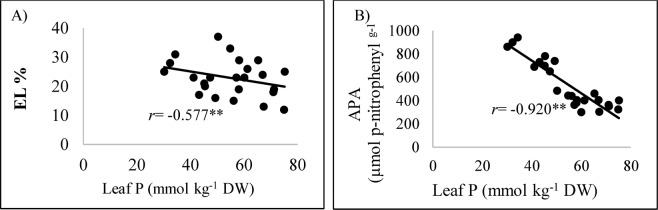
Correlation of leaf phosphorus (P) with electrolyte leakage [EL; (**A**)] and acid phosphatase **[**APA; (**B**)] enzyme activity in the leaves of maize plants grown under phosphorus and water deficiency stresses applied individually or in combination with or without soil amendments. **: correlations are significant at *P* ≤ 0.01.

One of the main stress-induced cell problems is impaired membrane integrity and permeability. An increase in electrolyte leakage, which reflects a loss of ability of biological membranes to regulate the transport of ions, has been reported under drought stress, e.g. in kochia (*Kochia scoparia* (L.) Schrad.) by Masoumi, *et al*.^92^ and in garden huckleberry (*Solanum scabrum* Mill.) by Assaha, *et al*.^93^. As phosphorus is directly linked with energy storage and ATP formation in plants^94^, thus its deficiency can impair membrane transport mechanisms and reduce plant growth, particularly under water deficit regimes^92^, and similarly in the current experiment, there was a significant (*P* ≤ 0.01) nonlinear correlation (*r* = −0.577) between leaf P content and EL of maize plants (Fig. 7A). Soil amendments with S or leonardite + humic acid significantly enhanced chlorophyll contents and improved membrane and PSII integrity in maize plants subjected to both stresses applied singly or jointly. The latter can be attributed to the reduced P fixation and increased soil P content that is supported by the increased leaf P in the maize plants treated with SAs.

### Effect of phosphorus deficiency, water stress and their combination, and S-enriched amendments on water status parameters

Under water and P deficiency stress conditions, RWC decreased significantly. It has been reported that low RWC is generally associated with stomatal closure, thereby leading to reduced CO_2_ availability and hence reduced rate of photosynthesis and impaired antioxidant/reactive oxygen species balance^83,95,96^. In the current experimentation, reduced chlorophyll content and *Fv/Fm* can be associated with low RWC in the maize plants exposed to both P deficiency and water stress. Water stress and P deficiency also significantly affected Ψw. Usually, RWC was found to be positively associated with leaf water potential (Ψw). For example, water stress reduced leaf water potential and RWC in parallel in soybean^97^. Leaf or tissue water potential (Ψw) is frequently used as a prospective selection criterion of plant stress tolerance^98^, because the reduction in Ψw caused by enhancement in hydraulic stress leads to reduction in photosynthetic CO_2_ assimilation^99,100^. Although leaf water potential of the maize plants suppressed markedly under both stresses, RWC decreased more prominently when those were applied in combination. Soil amendments with SAs in SA1 and SA2 treatments improved RWC and leaf water potential effectively, particularly when combined stresses were applied. Abuelsoud, *et al*.^101^ reported that sulfur, in addition to be an important macronutrient, participates in sulfur-containing compounds playing a critical function in osmotic adjustment in plants exposed to water deficit conditions. Therefore, it is still unclear in our study whether the effect of S or other factors in leonardite and humic acid applied soils were responsible for enhanced stress tolerance in the maize plants subjected to SAs amended soils.

### Effect of phosphorus deficiency, water stress and their combination and S-enriched amendments on leaf P concentration and acid phosphatase activity

In alkaline clay soils, solubility of P is among the main causes of P deficiency and reduced crop production^102,103^. One of hypotheses in the present experiment was to test whether S-enriched SAs would affect solubility of P and plant P uptake in alkaline clay soil. Here, application of SAs significantly increased tissue P levels in the P stressed maize plants. As supported by Khan, *et al*.^39^, application of sulfur fertilizers likely increases the P availability in calcareous soils. This is because, sulfur supplementation to the soil results in the generation of H_2_SO_4_, which in turn slightly reduces soil pH and increases solubility of P^104^. Similarly, an increase in P uptake, plant growth and yield were found due to exogenously applied S-enriched leonardite under clay and loamy sand soils^105^. In this study, we did not monitor the soil pH, therefore, it was not clear at what extent change in soil pH was responsible for the observed positive effects after application of S-enriched humic acid and leonardite. However, the present study clearly showed that in alkaline clay soil such additives can alleviate P deficiency-induced adverse effects on plants via enhancing maize P uptake.

Acid phosphatase activity (APA) is one of crucial traits because of its substantial role in plant P utilization^106,107^. The correlation between the APA activity and leaf P was significant (*P* ≤ 0.01) (*r* = −0.920) (Fig. 7B), indicating that when plants are subjected to P deficiency, APA activity increases to utilize more P. Wasaki, *et al*.^108^ have also shown that APA activity is one of the important plant traits indicating P deficiency.

### Effect of phosphorus deficiency, water stress and their combination, and S-enriched amendments on peroxidation

Free radicals generated by oxidative stress leads to lipid peroxidation thereby causing membrane deterioration in plants^88^. Peroxidation is frequently considered as the most damaging cellular response to stress conditions and is sometimes considered as an indicator of stress severity^109^. In the present study, water stress as well as phosphorus deficiency increased the MDA and H_2_O_2_ contents significantly. Excess accumulation of H_2_O_2_ can impair the cell redox potential and may lead to increased levels of antioxidants resulting into the alteration of antioxidant system^110^. In the current experimentation, soil amendments with leonardite and humic acid significantly reduced the P deficiency and water deficit induced oxidative stress. Of both amendments, SA2 was found to be more effective compared to SA1 in reducing both MDA and H_2_O_2_ contents in the maize plants under all stress conditions. Similarly, in a previous study with maize plants, application of HA was found to be very effective in reducing lipid peroxidation^48^. Moreover, humic acid has been shown to be effective in improving stress-induced lipid peroxidation in maize^111^.

### Effect of phosphorus deficiency, water stress and their combination, and S-enriched amendments on antioxidant enzymes

Under water deficit conditions, ROS generally accumulate due to imbalanced ROS/antioxidant activity ratio^87,88^. Under stress situation, enzymatic antioxidants such as SOD, POD and CAT play a key role in preventing ROS damage^112–114^. In the current investigation, the activities of these enzymes increased significantly under P deficiency. Application of SAs as SA1 and SA2 treatments effectively increased the activities of enzymatic antioxidants including those of SOD and CAT more dominantly under water deficit conditions. The latter indicates that the putative function of SAs-induced water deficit resistance in the maize plants may be due to their roles in reinforcing the antioxidant defence systems to nullify more H_2_O_2_ which maintained the leaf chlorophyll content.

## Conclusions

The present study clearly showed that combined stress (water stress and P deficiency) caused a considerable decrease in maize yield and yield related traits such as *F*_v_*/F*_m_, chlorophyll content, and leaf relative water content. However, supply of S-enriched LEO and HA as soil amendments mitigated the negative effects of both stress factors and increased plant growth, and yield. Our data clearly showed that addition of SAs specifically increased the antioxidative defense system and photosynthetic machinery of maize plants under water stress and P deficiency. Therefore, application of S-enriched leonardite and humic acid can be recommended for field application under water limited calcareous soils.
